# Understanding of Physical Activity in Social Ecological Perspective: Application of Multilevel Model

**DOI:** 10.3389/fpsyg.2021.622929

**Published:** 2021-03-05

**Authors:** Yoongu Lee, Sanghyun Park

**Affiliations:** ^1^Department of Sports Science, Sun Moon University, Asan, South Korea; ^2^Department of Sport Industry Studies, Yonsei University, Seoul, South Korea

**Keywords:** CHS, social ecological model, physical activity, individual characteristic, social environment, physical environment

## Abstract

In the social ecological model, personal characteristics are important determinants of health behaviors, however, multi-dimensional approaches that consider social and physical environments must be utilized to gain a broader picture. Accordingly, this study examines the effects of personal, social, and physical environment variables as factors affecting levels of physical activity (METs). Our findings are based on 72,916 responses from the 2015 Community Health Survey in South Korea. Individual characteristics considered included sex, education level, marital status, age, and income. The social environment variables considered were trust between neighbors and the social network with neighbors. The physical environment variables were satisfaction with living environment and satisfaction with public transportation. The analysis was conducted using a multilevel model in order to accurately consider the characteristic differences of the variables. Regarding personal characteristics, sex, education level, and age have a significant effect on physical activity. Of the social and physical environment variables, social network with neighbors and satisfaction with public transportation have a significant effect on physical activity. This study confirms that a macroscopic understanding is needed to explain individual levels of physical activity; the results of this study will be helpful for public health interventions concerning physical activity.

## Introduction

Engagement in physical activity is widely known to be beneficial to human health in many ways. However, in spite of the range of positive benefits to physical activity, the proportion of adults in Korea who do not achieve sufficient levels of physical activity increased from 24.6% in 2008 to 42.9% in 2014 (Yang, 2016). The effects on society of neglecting physical activity, including the resulting deaths, are greater than those of obesity and similar to those of smoking (Lee et al., 2012).

Increasing levels of physical activity has been a goal in many public health contexts and theoretical studies have been performed to predict engagement in physical activity. The social ecological model constructed by Bronfenbrenner (1977) proposed that behavior is affected by a range of variables on the individual level and the broader social, physical, and policy environments. Variables on the individual level can include demographic characteristics, as well as beliefs and attitudes in relation to behavior. Factors of social environment consider how supportive of engagement in physical activity the people around an individual are. Physical environment factors include the facility accessibility of engagement in physical activity. Finally, the policy environment describes the laws and policies of the central and local governments for engagement in physical activity. Theories such as Social Cognitive Theory (SCT), Theory of Planned Behavior (TPB), Self-Determination Theory (SDT), and Transtheoretical Model (TTM) for explaining physical activity greatly enhance understanding of psychological motivators (Nigg et al., 2008; Buchan et al., 2012). However, the social ecological model puts forward a multidimensional approach, incorporating the social and physical environment as well as admitting that personal aspects are important factors in health behavior (McLeroy et al., 1988). Furthermore, although personal, social, and physical environments are all quite important for promoting physical activity, such factors may have synergic effects (Susser and Susser, 1996). This is supported by the observation that it is more efficient to improve the environment around individuals than to directly change the individuals themselves (Spence and Lee, 2003).

A study by Wilson et al. (2004), which described the socio-economic level of individuals in the social ecological model, found that people who have a lower socio-economic status are less likely to engage in physical activity than those of higher socioeconomic status. Other studies have found similar results, presenting further complex links. Görner et al.’s (2020) study reports that socioeconomic factors affect the swimming ability of middle school students: middle and upper economic classes were found to better swimmers due to more learning opportunities and to access to better educational environments. Additionally, people with lower socio-economic statuses are less aware of their own health problems than those with higher statuses, resulting in reduced participation in physical activity (Prentice, 2006). It is widely acknowledged that sex, age, income, and psychological variables can predict physical activity (Hwang and Kim, 2017; Siahpush et al., 2019).

Recent studies (Bjornsdottir et al., 2019; Yen and Li, 2019) have investigated social and physical environment variables, which, unlike variables on the individual level, can be integrated into studies despite the lack of empirical research. Thornton et al. (2017) used the social ecological model to establish the effects of individual demographic and psychological variables, as well as social and physical environmental variables, on physical activity, indicating that all social ecological variables have significant effects. Neighborhood social cohesion, considered an important facet of social environment, is one of the most important factors in promoting health awareness. Conceptually, it considers the degree of trust, familiarity, and strength of network relations with neighbors (Mendes de Leon et al., 2009). Furthermore, Neighborhood Social Cohesion has been verified as an important variable affecting the physical activity of the elderly (Kim et al., 2020). In addition, studies into physical environment prove that the architectural and urban environment of the local community influences physical activity (Sun et al., 2020). To create a physical environment conducive to promoting physical activity various factors should be considered, such as pedestrian infrastructure, public transport access and infrastructure, and access to community and park facilities (Omura et al., 2020). These studies show how important it is for an individual to have people around who are also engaged in exercise and to provide support for the individual’s physical activity, underlining that infrastructure and accessibility play a significant role in facilitating engagement in physical activity. Moreover, other studies, such as those of de Bruijn et al. (2006) and Lee and Shepley (2012), have integrated physical environmental variables with the planned behavior model theory to investigate physical activity levels. This work has proved that the physical environment must be accounted for in promoting physical activity. As previously noted, studies have worked to research physical activity by integrating the social ecological model with other theoretical models.

Nevertheless, it should be acknowledged that the studies that have applied this social ecological model show certain limitations in their analytical methods. The individual, social, and physical environments, considered as variables of a social ecological model, exist within a multilevel structure. To expand upon this: if variables related to individual characteristics should be assigned level 1 variables and social and physical environment variables, which can be considered group characteristics, should be assigned level 2. Nevertheless, in the studies of de Bruijn et al. (2006) and Rhodes et al. (2006) the relationship between physical environment variables and physical activity was verified using a structural equation model and path analysis. Nategh et al.’s (2017) study verified social ecological variables through the Pearson correlation test. The multilevel model can be used to analyze the data divided into individual and group information but the methods used in previous studies could result in interpretation errors because the analysis neglects such levels of variables. For this reason, this study adopts the multilevel structure outlined above. The Community Health Survey calculates the basic data and statistics necessary for establishing a national health promotion plan and a local health care plan at the national level. Using this data has many advantages: not only is it collected through a systematic process but it produces more reliable results because of its large sample. A study by Koo and Kim (2018) used the Community Health Survey data to derive social ecological factors affecting physical activity levels of the elderly. In this study, the interaction effect was analyzed through decision tree analysis. Kim and Suh (2017) analyzed the social ecological factors affecting the walking habits of office workers using a multilevel model. Accordingly, this study aimed to investigate the social ecological factors affecting both moderate and vigorous physical activity as well as walking. In addition to conventional identification of the determinants of physical activity on the individual level, a macroscopic investigation through the social ecological model could help provide a more persuasive view of human behavior. Thus, this study drew on panel data from the Community Health Survey in South Korea, assessing them through the social ecological model. Specifically, this study investigated the effects that individual characteristics and social and physical environments exert on individual physical activity. Thus, this study utilized data from the 2015 Community Health Survey in South Korea and set the research questions below to achieve its objectives.

1. How do individual characteristics (sex, education level, marital status, age, and income) affect physical activity?
2. What effects do social (neighborhood trust and social network with neighbors) and physical (life environment satisfaction, and public transportation satisfaction) variables have on physical activity?

## Materials and Methods

This study investigated the effects that individual characteristics and social and physical environments have on individual physical activity. Specifically, this study used a multilevel model to analyze level 1 individual characteristic variables that affect physical activity, including sex, education level, marital status, age, and monthly income, and level 2 social environment variables (trust between neighbors and social network with neighbors) and physical environment variables (life environment satisfaction and mass transportation satisfaction) to statistically verify the effects of variables on individual physical activity levels.

### Characteristics of the Analysis Data

**FIGURE 1:**
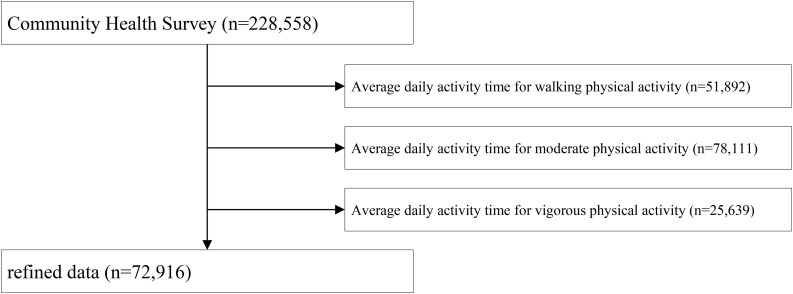
Flow chart.

The data used in this study was obtained from the 2015 Community Health Survey of Korea Centers for Disease Control and Prevention, which is a 2015 panel data survey of indicators related to residence and health for adults aged over 19 covering 17 cities and provinces in South Korea. Data collection was coordinated by the Korea Centers for Disease Control and Prevention. The acquisition of the raw data for research purposes was approved by the commission board after undergoing a privacy and research ethics process. The raw data is representative because it was extracted using the weight of population structure in South Korea. Although the raw data included responses from 228,558 people, 72,916 people were selected for the final analysis through a data-refining process. To calculate metabolic equivalents (METs) we had to use variables regarding individual activity time. However, these variables had a lot of missing values. For instance, a variable with regard to average daily activity time for walking physical activity (minutes) had 51,892 missing values and a variable regarding average daily activity time for moderate physical activity (minutes) had 78,111 missing values. Therefore, we used list wise deletion as we still had adequate data to represent the population after removing 155,642 observations with missing values (see Figure 1). Table 1 presents the general characteristics of the subjects.

**TABLE 1:** Demographic characteristics of analytical data.

	Type	*n*	%
Sex	Male	35,957	49.3
	Female	36,959	50.7
Education level	Uneducated	3,483	4.8
	Village school	118	0.2
	Elementary school	11,712	16.1
	Middle school	7,864	10.8
	High school	21,130	29.0
	College	8,510	11.7
	University	17,129	23.5
	Graduate school	2,970	4.1
Marital status	Married	52,055	71.4
	Divorce	2,285	3.1
	Bereavement	6,226	8.5
	Separation	859	1.2
	Single	11,491	15.8
Age	19	810	1.1
	20–29	7,447	10.2
	30–39	10,891	14.9
	40–49	14,840	20.4
	50–59	15,431	21.2
	60–69	12,475	17.1
	70–79	9,031	12.4
	80–89	1,933	2.7
	90-	58	0.1
Monthly income (unit: ￦1,000)	-500	4,183	5.7
	500–1000	8,394	11.5
	1000–2000	12,287	16.9
	2000–3000	13,944	19.1
	3000–4000	12,326	16.9
	4000–5000	8,726	12.0
	5000–6000	5,251	7.2
	6000-	7,805	10.7

### Dependent Variables

Physical activity, which appears in this study as a dependent variable, was calculated using the International Physical Activity Questionnaire (IPAQ), following previous studies (Oh et al., 2007). The amount of physical activity engaged in is presented in terms of METs, calculated from the answers to the IPAQ. The Community Health Survey provided open-ended questions designed to find the number of days and hours consumed by walking (low-impact), moderate, and vigorous physical activity during the previous week. Individual METs were assigned to subjects based on the following formulas.

METs = Vigorous MET + Moderate MET + Walking MET
  - Vigorous MET = 8 × average daily activity time for vigorous physical activity (minutes) × number of days performed
  - Moderate MET = 4 × average daily activity time for moderate physical activity (minutes) × number of days performed
  - Walking MET = 3.3 × average daily activity time for walking physical activity (minutes) × number of days performed

### Independent Variables

The level 1 independent variables (individual characteristic variables) used in this study included sex, education level, marital status, age, and monthly income. To provide practical meanings to these variables for analysis, all individual characteristics excluding age were converted into dummy variables (reference group: male, below high school, married, and below 1,000 thousand won). These reference groups were only used for statistical comparison.

Level 2 variables (environmental characteristics) included social environment (trust between neighbors and social network with neighbors) and physical environment (life environment satisfaction and mass transportation satisfaction) as independent variables. Statements were given to measure the satisfaction levels of each independent variable. Level of trust between neighbors was measured through the item “I can trust the people in my neighborhood,” and the social network with neighbors item asked, “How often do you see or contact your neighbors who are most frequently in contact.” The life environment satisfaction item was “I am satisfied with life environment in my neighborhood (including electricity, water and wastewater system, garbage collection, and sports facilities),” and the mass transportation satisfaction item was “I am satisfied with mass transportation status in my neighborhood (including bus, taxi, subway, and train).”

Except for social network with neighbors which was measured by 7-point Likert scale, the items for trust between neighbors, life environment satisfaction, and mass transportation satisfaction were set as categorical variables (Yes/No). These items were assessed with the percentage of respondents who selected “Yes” in each region. For example, of the 6,652 respondents in the Seoul region, 4,195 respondents answered Yes to the item “I can trust the people in my neighborhood.” Therefore, 63.0% was recorded as the value for the variable. These variables were assessed by two experts to ensure the content validity. Table 2 summarizes specific information about the variables.

**TABLE 2:** Variables.

DV	METs	Calculated by the METs formula
IV	Sex	Dummy variable
	Education level	Dummy variable
	Marital status	Dummy variable
	Age	Original scale
	Monthly income	Dummy variables
	Trust between neighbors	Percentage of participants who response “Yes”
	Social network with neighbors	Average value (7-point Likert Scale)
	Life environment satisfaction	Percentage of participants who response “Yes”
	Mass transportation satisfaction	Percentage of participants who response “Yes”

### Data Processing

The data obtained from the Community Health Survey were cleaned using SPSS 24.0 and frequency analysis was conducted to find basic information on the 72,916 respondents for the final analysis. A multilevel model analysis was then conducted utilizing HLM version 8 (Raudenbush and Bryk, 2002). Model 1 is an unconditional model with no independent variables and is the base model for calculating ICC only. Model 2 considers variables at the individual level, which attempts to explain physical activity only using individual characteristics. Model 3 considers group-level variables (social and physical environment) alongside Model 2 and is the model that is ultimately tested in this study. In other words, Model 3 applies a social ecological model that considers individual and environmental variables. The reason for the three models is to calculate the ICC (intra-class correlation coefficient) for each model. ICC can be theoretically meaningful in understanding how much of the overall variation in the response is explained simply by clustering (Woltman et al., 2012). We used intra-class correlation analysis (ICC) to compare the variance between between-group and within-group. ICC was calculated using the following equation τ_*00*_ is the averaged variance between groups.

$$ICC=\frac{\tau_{00}}{\tau_{00}+\sigma^{2}}$$

## Results

Table 3 shows the results of the multilevel analysis on level 1 and level 2 variables. Based on a comprehensive review of the level 1 variables, males were more likely to participate in physical activity than females (*t* = 12.2^∗∗∗^). Regarding education level, the lower the educational level, the more physical activity was recorded (*t* = 16.4^∗∗∗^). Finally, it showed that physical activity decreases with increasing age (*t* = −4.2^∗∗∗^). However, the differences in levels of physical activity according to marital status and monthly income were not statistically significant.

**TABLE 3:** Results of multilevel analysis.

Parameter		Model 1 null model			Model 2 random intercept model			Model 3 intercept as outcome model		
		Estimate	*SE*	*t*	Estimate	*SE*	*t*	Estimate	*SE*	*t*
Level 1	Sex (male)				1136.1	42.6	26.6***	1135.8	92.7	12.2***
	Education level (below high school)				1952.8	53.5	36.4***	1951.0	118.3	16.4***
	Marital status (married)				148.8	50.3	2.9**	149.1	117.3	1.27
	Age				–12.6	1.73	−7.2***	–12.6	2.9	−4.2***
	Monthly income (1000–5000)				78.4	65.4	1.1	78.9	176.7	0.4
	Monthly income (5000-)				–317.0	82.8	−3.8***	–314.0	200.7	–1.5
Level 2	Trust between neighbors							–49.6	60.2	–0.8
	Social network with neighbors							1302.0	563.2	2.3*
	Life environment satisfaction							43.4	41.7	1.0
	Mass transportation satisfaction							66.7	12.2	5.4***
ICC		0.03			0.03			0.02		
Deviance		1469838.6			1467631.7			1467571.7		

*p < 0.05, **p < 0.01, ***p < 0.001.

Amongst the level 2 variables, better social networks with neighbors resulted in more physical activity (*t* = 2.3^∗^). Additionally, the higher the level of mass transportation satisfaction, the more physical activity was found (*t* = 5.4^∗∗∗^). On the other hand, trust between neighbors and life environment satisfaction had no statistically significant effects on physical activity. The results suggest that the stronger the social network with neighbors and the greater the satisfaction mass transportation, the more physical activity there is. The value of intra-class correlation was determined as 0.03 or less, which suggests that the differences in physical activity are more important in relation to the individual level than the environmental level (Koo and Kim, 2018).

Comprehensively looking at the results, it was found that education level was the factor that had the greatest influence on physical activity in level 1 variables representing individual characteristics. In the level 2 variable, which is an environmental characteristic, mass transportation satisfaction has the greatest effect on physical activity, suggesting that access to physical activity facilities is the most important. In addition, as variables explaining physical activity, social and physical environments are also important, but personal characteristics factors are more important than anything else.

## Discussion

The social ecological model contributes significantly to the theoretical understandings of engagement in physical activity. This study utilized a multilevel model to describe the individual characteristic variables and the social and physical environment variables that affect individual physical activity levels. Thus, the results of this study form a contribution to the understanding of the determinants of an individual’s engagement in physical activity from a macroscopic viewpoint.

Sex, education level, and age all had a significant effect on physical activity. Males reported statistically higher physical activity than females. A study by Li et al. (2017) on the elderly reports that males walk more steps per day than females do and that males engage in more intense physical activities than females. A study focusing on college students by Lauderdale et al. (2015) indicates sex differences in physical activity using self-determination theory. That study reported that male students engaged in more physical activity than female students due to their higher intrinsic motivation. Higher motivation implies more frequent execution of an action due to its inherent interest and enjoyability, so this result could be explained by the observation that male students are more interested in physical activity than female ones. Nevertheless, because that study was focused on college students, its results should be interpreted with care. Eime et al. (2018) have found that females are less likely to participate in physical activity than males. They suggested increasing participation by modifying and improving motivations to be physically active. Females are afraid of injuries and are less likely to participate as they perceive the benefits to health as low (Nxumalo and Edwards, 2017). In order to improve the perception of physical activity amongst females, physical activity needs to be better promoted to women and there needs to be more programs designed for them.

The lower the educational level, the higher the level of physical activity. Our results are in line with those of Bauman et al. (2011) and Wallmann-Sperlich et al. (2013), whose studies indicate that people with higher levels of education tend to engage in more sedentary behavior. That is, people with relatively higher levels of education are more likely to have office jobs that require more sedentary activity and they tend to move from place to place by car, overall seeking to reduce the level of their physical labor. This could explain the results of this study. However, participation in vigorous activities such as swimming and jogging is subject to financial, social, and environmental constraints and a low education level may still hinder participation. In addition, a longitudinal study spanning 13 years showed that the inequality gap concerning vigorous activity increased over time (Gugusheff et al., 2020). In order to remove socioeconomic barriers to access for different types of physical activity, environments and policies that make it possible to participate in sports at a low cost should be supported. On the other hand, it was found that income was not a factor affecting physical activity. There may be differences in the type of physical activity depending on income, but a low income does not cause a lack of physical activity. According to Shuval et al. (2017), low income is positively related to light activity and high income is related to less frequent and stronger activity. In other words, there may be differences in physical activity patterns depending on income but there is no difference in physical activity levels (METs). In addition, parks and facilities in South Korea are continuously being developed and allow anyone to participate in physical activities regardless of cost. Physical activity decreased as age increased. This result is consistent with numerous previous studies and a multi-faceted approach is required if physical activity levels of the elderly are to be increased (Laitakari et al., 1996; Cheung et al., 2007). Netz and Raviv (2004) attributed the reduced physical activity of the elderly to their lower self-efficacy related to physical activity. This creates a barrier to entry, limiting access to physical activity. Intervention is necessary and a macroscopic approach is required. Telama et al. (2005) reported that continuous exercise in adolescence can increase the likelihood of exercise in old age. In that study, incentive systems to enhance physical activity in adolescence and promote continuous exercise schedules were explored. More detailed policies and research related to strategies for enhancing physical activity should be developed on a life-cycle basis.

The level 2 variables that relate to social and physical environments show that the social network with neighbors and mass transportation satisfaction had a significant effect on physical activity. Greater social networks between neighbors imply higher frequency of direct contact. Social isolation can significantly reduce physical activity in adults, meaning that close networks of people nearby constitute a key factor in enhancing physical activity (Caspi et al., 2006). A study of Australian 1,289 adults from 18 to 89 years old (Lauder et al., 2006) showed that isolated people tend to be more overweight than socially connected people. The benefits of social networks include the promotion of social comparison and enhanced self-efficacy through encouragement by others (Duncan and McAuley, 1993; Rovniak et al., 2002). Social networks are beneficial in increasing people’s level of physical activity as they have a positive relationship with physical activity (Aliyas, 2020). In addition, social networks minimize the disturbance and stress factors of health behaviors (Gao et al., 2015). Help from neighbors can aid in finding solutions to disturbance factors through discussion of countermeasures.

Mass transportation satisfaction as a physical environment variable has a positive effect on physical activity. The physical environment includes sports facilities, mass transportation facilities, and access to facilities in the regional community. Among these factors, mass transportation has been found to contribute positively to individual health, in addition to its other benefits. People using mass transportation walk 5–33 min more than those using a car, and the level of physical activity among those people using mass transportation is higher, for that and other reasons (Besser and Dannenberg, 2005; Wasfi et al., 2013; Lachapelle and Pinto, 2016). Well-equipped sports facilities in the regional community are an important variable that can affect physical activity. This study found that mass transportation satisfaction has a positive effect on physical activity, suggesting that it can, among other things, improve accessibility to sports facilities.

In spite of the valuable contributions it offers, this study has some limitations. The age of the participants in the analysis of this study ranged from 19 to 70 years, meaning that the results should be carefully examined to enable the development of intervention programs for specific age groups. Furthermore, studies on specific age groups should be performed to develop specific intervention measures. In addition, because the data in this study was drawn from limited measurement variables through the Community Health Survey data, follow-up work could build upon these findings and obtain more detailed information by examining additional variables through additional surveys. Finally, Community Health Survey participants that did not respond to physical activity intensity items were excluded from the analysis as METs could not be calculated for this group. While the sample completing the physical activity items is not notably biased toward any particular group, demographic distributions do differ from the full survey sample, which may limit generalizability to some extent.

## Conclusion

On an individual level, males are more likely to engage in physical activity, which suggests a gendered difference in motivation to exercise. As such, the issue of how to enhance physical activity levels among females requires closer consideration. In relation to education level, those with more education tend to engage in physical activities less, which may be related to the fact that those with higher levels of education more commonly work in office-based jobs. To combat the reduced physical activity that comes with aging it is necessary to promote continuous participation in exercise from adolescence, lowering the barrier to engagement in physical activity at older ages. Social and physical environments should be enhanced to enable more physical activity within communities by organizing leadership-level meetings and clubs that facilitate local social networks and promoting the development of mass transportation on the regional community level.

## Data Availability Statement

The original contributions presented in the study are included in the article/supplementary material, further inquiries can be directed to the corresponding author/s.

## Ethics Statement

Ethical review and approval was not required for the study on human participants in accordance with the Local Legislation and Institutional Requirements. Written informed consent for participation was not required for this study in accordance with the National Legislation and the Institutional Requirements.

## Author Contributions

Both authors contributed to literature review, study design, data analysis, drafting, and revising.

## Conflict of Interest

The authors declare that the research was conducted in the absence of any commercial or financial relationships that could be construed as a potential conflict of interest.
